# Prioritization of Features for Mobile Apps for Families in a Federal Nutrition Program for Low-Income Women, Infants, and Children: User-Centered Design Approach

**DOI:** 10.2196/30450

**Published:** 2021-07-30

**Authors:** Summer J Weber, Elyse Shearer, Shelagh A Mulvaney, Douglas Schmidt, Chris Thompson, Jessica Jones, Haseeb Ahmad, Martina Coe, Pamela C Hull

**Affiliations:** 1 Markey Cancer Center University of Kentucky Lexington, KY United States; 2 College of Agriculture Tennessee State University Nashville, TN United States; 3 School of Nursing Vanderbilt University Nashville, TN United States; 4 School of Engineering Vanderbilt University Nashville, TN United States; 5 233 Analytics Nashville, TN United States; 6 Meharry-Vanderbilt Alliance Meharry Medical College Nashville, TN United States

**Keywords:** WIC, mobile technology, maternal-child health, childhood obesity, nutrition, government programs, mobile app, user-centered design, low income, women, infant, child, formative, development

## Abstract

**Background:**

The Special Supplemental Nutrition Assistance Program for Women, Infants, and Children (WIC) is a federal nutrition program that provides nutritious food, education, and health care referrals to low-income women, infants, and children up to the age of 5 years. Although WIC is associated with positive health outcomes for each participant category, modernization and efficiency are needed at the clinic and shopping levels to increase program satisfaction and participation rates. New technologies, such as electronic benefits transfer (EBT), online nutrition education, and mobile apps, can provide opportunities to improve the WIC experience for participants.

**Objective:**

This formative study applies user-centered design principles to inform the layout and prioritization of features in mobile apps for low-income families participating in the WIC program.

**Methods:**

To identify and prioritize desirable app features, caregivers (N=22) of the children enrolled in WIC participated in individual semistructured interviews with a card sorting activity. Interviews were transcribed verbatim and analyzed using constant comparative analysis for themes. App features were ranked and placed into natural groupings by each participant. The sum and average of the rankings were calculated to understand which features were prioritized by the users. Natural groupings of features were labeled according to participant descriptions.

**Results:**

Natural groupings focused on the following categories: clinics/appointments, shopping/stores, education/assessments, location, and recipes/food. Themes from the interviews triangulated the results from the ranking activity. The priority app features were balance checking, an item scanner, and appointment scheduling. Other app features discussed and ranked included appointment reminders, nutrition training and quizzes, shopping lists, clinic and store locators, recipe gallery, produce calculator, and dietary preferences/allergies.

**Conclusions:**

This study demonstrates how a user-centered design process can aid the development of an app for low-income families participating in WIC to inform the effective design of the app features and user interface.

## Introduction

The Special Supplemental Nutrition Program for Women, Infants, and Children (commonly known as WIC) is one of the most successful [1] and cost-effective [2,3] nutrition programs aimed at improving maternal, infant, and child health in the United States. WIC is a federal program that provides nutritious food (eg, fresh and frozen produce, whole grains, milk, eggs), nutrition education, and health care referrals to low-income women during pregnancy and the postpartum period and to their children from birth up until their fifth birthday. WIC’s services have been monitored and researched for decades and its contribution has been attributed to a myriad of positive health outcomes for its participants.

For example, better birth outcomes with WIC are well documented [4] and include beneficial interaction effects on birth weight [5-7] and significantly reduced probability of highly premature births [5]. Nutrition-related outcomes include—but are not limited to—positive changes in dietary intake [8], healthy food purchases [9], and reductions in obesity [10]. WIC has also been associated with improvements in household food security [10], childhood immunizations [11,12] and health care utilization [11].

Despite all the positive effects of WIC, barriers to the usage of the program and participation exist at the interpersonal (eg, family support), institutional (eg, prohibitive work schedules), clinical (eg, long wait times), shopping (eg, WIC items that are hard to find, not in stock, and inconsistent within and between vendors), and administrative/systems levels (eg, restrictive benefits and stigma surrounding government assistance) [13-17]. Addressing the issue of early exits from the WIC program has been identified as one of the key areas in a research needs assessment put forth by the National WIC Association (NWA) [18], and a national program retention campaign was rolled out in 2018 [19].

Technologies to improve and streamline the WIC experience for participants have been offered as a solution at the clinic and shopping level. These technologies have become particularly essential after the onset of the COVID-19 pandemic. In many states, participants can complete web- or app-based nutrition education in their own time, rather than attending in-person classes at the clinic to fulfill some of their nutrition education requirements [20-22].

At the shopping level, WIC state agencies were mandated to switch from a paper voucher system to an electronic benefits transfer (EBT) system for WIC by 2020, with some waivers of extension [23] in which participants make WIC purchases using an EBT card that resembles a debit card. Implementation of WIC EBT (or electronic WIC [eWIC]), as many state agencies refer to it) is a considerably large administrative undertaking that stands to benefit participants considerably owing to increased ease of use at checkout, elimination of the risk of theft or loss of paper vouchers, increased redemption of benefits, and decreased stigma associated with purchasing food using a discreet benefit card rather than cumbersome paper vouchers that can cause difficulties for customers and cashiers [14,24-26].

Although beneficial, modernization of the redemption process by eWIC comes with certain challenges for the participants in lieu of paper voucher usage. In the recent past, participants could utilize their paper vouchers (also called food instruments) to view and keep track of itemized WIC benefits, quantities, and sizes according to their food prescription. Without a source of guidance, shoppers may feel unclear about their eWIC benefit balance, especially if they are new to the program and unfamiliar with WIC foods and prescription changes that occur as children age or when women transition from pregnancy to the postpartum period. WIC families also face the same barriers when choosing the correct items while shopping.

A potential solution to the challenges outlined above are mobile apps that provide WIC families with balance checking features and barcode scanners to verify eligible WIC items [27-29]. The study described in this paper builds off a project that informed the development of a prototype mobile app called Children Eating Well (CHEW) designed to support WIC families prior to eWIC transition [28]. The objective of the current study was to conduct interviews with caregivers of WIC- enrolled children (referred to as “WIC caregivers”) to inform the design and prioritization of the features for an enhanced version of the CHEW mobile app and other mobile apps for low-income families participating in the WIC program.

Although apps for WIC participants exist in many states [27], research studies on WIC apps are relatively sparse. A recent study found that usage of the most widely available commercial WIC app (WICShopper) was associated with higher levels of WIC benefit redemptions among WIC households in West Virginia [29]. Literature searches revealed only one previously published study that employed user-centered design principles to inform the development of WIC apps; that study focused on nutrition education and health behavior [30]. The objective of the current study was to conduct formative research applying user-centered design principles to inform the layout and prioritization of the features in mobile apps for low-income families participating in the WIC program that could target outcomes such as WIC family benefit redemption, diet quality, and obesity risk factors among preschool-aged children [31-33].

## Methods

### Recruitment

Caregivers/parents of children aged 2-4 years enrolled in WIC were recruited via fliers and posters at WIC clinics and health departments throughout Nashville, Tennessee, to participate in a 30-to-60–minute interview about mobile phone apps for WIC families. Participants were screened for eligibility via telephone. Eligible participants were 18 years or older, had a child aged 2-4 years enrolled in WIC, were the primary WIC shoppers in their households, and used a smartphone.

The interviews took place during July-August 2018, prior to the transition of the Tennessee WIC program to eWIC. Interviewees were informed at the beginning of the interview that WIC would be changing to eWIC over the course of the coming year. Therefore, the questions were framed around what features they would like to have in an app to use with eWIC.

### Study Procedures

A semistructured interview protocol was developed based on a multilevel model of factors influencing the perceived value of WIC [14], as well as formative research [28] and a review of existing WIC apps and features [27]. Using the semistructured interview protocol, participants were asked to speak about their experiences using WIC services, shopping for WIC foods, and using mobile phone technology. Participants were also asked to form mental models about using recipes, shopping lists, using WIC benefits, and checking their WIC balance.

**Figure 1:**
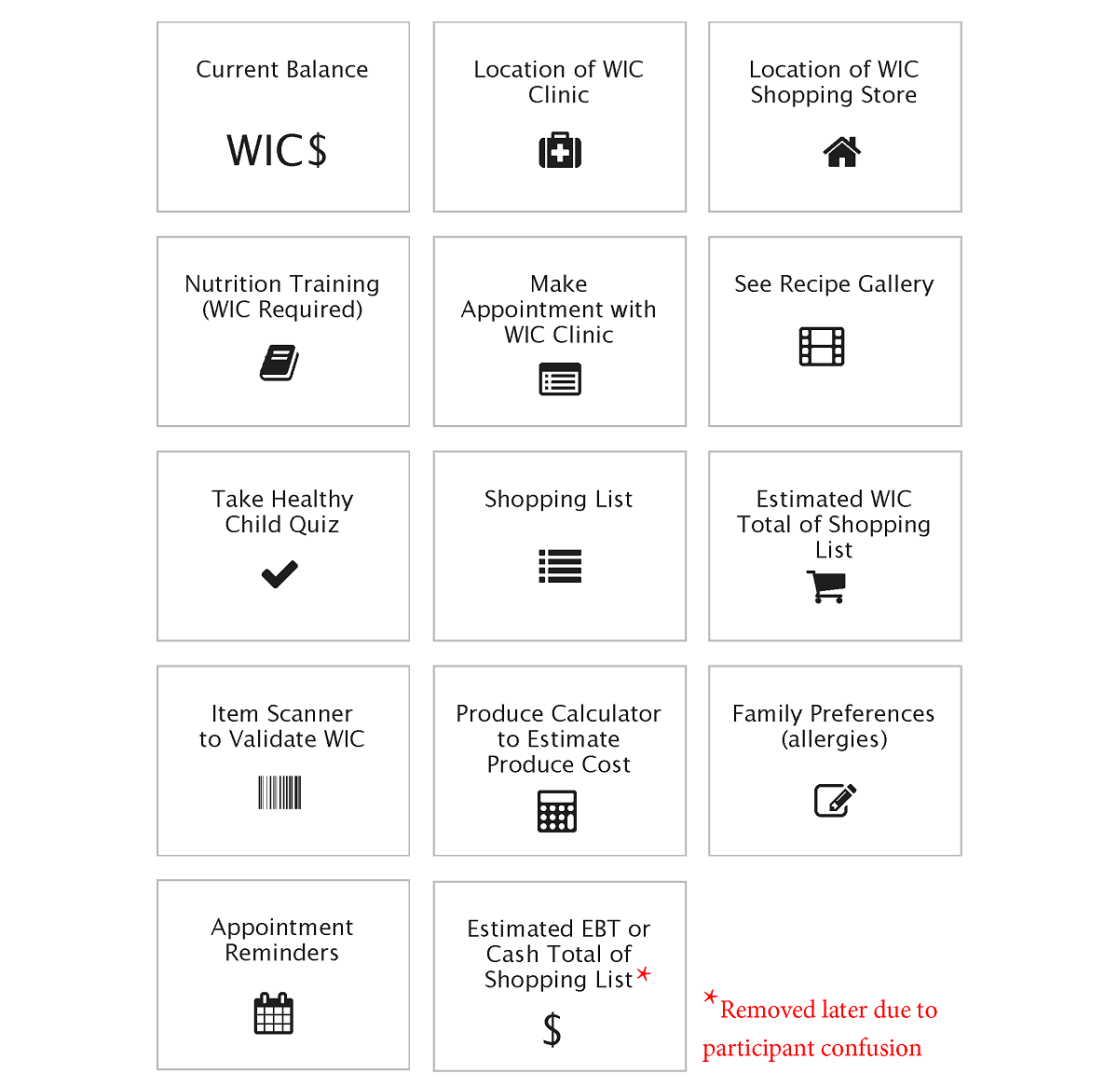
App feature activity cards given to WIC caregivers for sorting by level of importance and placing into natural groupings. WIC: Special Supplemental Nutrition Assistance Program for Women, Infants, and Children.

Potential in-app features were printed on 14 activity cards with icons to represent their function, as Figure 1 shows. Using a sorting activity, participants were first asked to sort the features by importance. They were then asked to place the cards into natural groupings of similar features. To record the prioritization of features and the natural groupings of features, photographs were taken during the card- sorting activity and labeled immediately following each interview.

### Quantitative Analysis

For the feature prioritization task, a numerical ranking from 1 to 14 (1 being most important and 14 being least important) was applied to each feature description for each participant. To comprehensively analyze the rankings, the sum and mean of each ranking were calculated. For the natural groupings task, the card groups in each photograph were circled and labeled according to the participants’ own descriptions, if any. A table of the card groupings and labels was created to track how participants would like to display and organize these features within an app.

### Qualitative Analysis

Interviews were transcribed verbatim, read several times to obtain a clearer understanding, coded, and then analyzed by constant comparative analysis [34] to identify themes, using the ATLAS.ti qualitative data analysis software (version 8.0, ATLAS.ti Scientific Software Development GmBH). A list of codes and code groups was updated and maintained throughout the analysis. Codes and code groups were queried via like groupings and then compared within and between transcripts.

The identification of themes was informed by previous research on barriers and facilitators to using WIC in general (eg, difficulties while shopping, clinic location) [13-17]. Analysis also focused on themes that were based on potential app features and tools for families who use WIC (eg, scanner, balance checking, recipes). Participants were recruited until the data reached saturation and no new information resulted from the interviews. The main themes were summarized, and sample quotes were selected that illustrated the themes.

Interrater reliability was determined according to the procedures of Gough and Conner [35]. Three additional coders completed code allocations to all 429 quotations, which were then checked to ensure a high level of correspondence (96.5%, 94.1%, and 92.3%, respectively). Lincoln and Guba’s criteria for trustworthiness of qualitative research [36] were applied to ensure credibility of the findings, which included the following:

- prolonged engagement (iterative interviews and correspondence with participants)
- persistent observation (staff and client interactions)
- peer debriefing (meetings and correspondence with the state agency and WIC site coordinators)
- triangulation of data (quantitative card sorting activity alongside qualitative interviews)
- negative case analysis (discussion of elements that contradicted patterns from the data)
- referential adequacy (archiving a portion of the data to be analyzed later to test the validity of the preliminary findings)
- member checks (verbal confirmations of accurate understanding conducted throughout the interviews and during follow-up interviews as part of the iterative process).

## Results

### Participant Characteristics

Table 1 shows the participant characteristics. All the interview participants were female except for 2 (WIC families’ primary shoppers are predominantly female). Of the 22 participants, 11 identified their race as Black or African American, 8 White, 1 Asian Indian, and 2 selected “other race.” Hispanic ethnicity was selected by 2 participants, and 6 participants reported that they were born in another country, which included Iraq (2), the Democratic Republic of Congo (2), Egypt (1), and India (1).

Just over half (12/22, 55%) of the participants were not married and had 1 or 2 children under the age of 18 years (13/22, 59%). Nearly one-third (7/22, 32%) of the participants also had an infant child aged below 2 years in addition to having 1 or more 2-to-4-year-old children. Most participants had high school degree or some college education (15/22, 68%).

**Table 1:** Characteristics of the Special Supplemental Nutrition Assistance Program for Women, Infants, and Children participants in Nashville, Tennessee, United States, determined via interviews (N=22).

Variable				Values, n (%)
**Gender**				
		Female	20 (91)	
		Male	2 (9)	
**Race**				
		Black or African American	11 (50)	
		White	8 (36)	
		Asian Indian	1 (5)	
		Other	2 (9)	
**Hispanic ethnicity**				
		Yes	2 (9)	
		No	20 (91)	
**Born in the United States**				
		Yes	2 (9)	
		No^a^	20 (91)	
**Marital status**				
		Married	10 (45)	
		Single-never married	5 (23)	
		Single-never married-lives with partner	4 (18)	
		Single-divorced	1 (5)	
		Refused to state	2 (9)	
**Number of children under 18**				
		1	3 (14)	
		2	10 (45)	
		3	6 (27)	
		4	1 (5)	
		5	1 (5)	
		9	1 (5)	
**Employment**				
		Full-time	8 (36)	
		Part-time	4 (18)	
		Not employed	10 (45)	
**Type(s) of WIC^b^** **packages received**				
	Pregnant woman		1 (5)	
	Breastfeeding woman		2 (9)	
	Partially breastfeeding woman		1 (5)	
	Postpartum (not breastfeeding) woman		2 (9)	
	Infant (0-23 months old)		7 (32)	
	Child (2-4 years old)^c^		26 (100)	
**SNAP^d^** **recipient**				
	Yes		9 (41)	
	No		13 (59)	
**Type of smartphone**				
	Android		13 (59)	
	iPhone		9 (41)	
**How often do you have your smartphone with you?**				
	Almost all the time		19 (86)	
	Often		2 (9)	
	Some of the time		1 (5)	

^a^ Participants not born in the United States were from Iraq (21 and 22 years in US), Egypt (9 years in US), the Democratic Republic of Congo (4 and 8 years in US), and India (23 years).

^b^WIC: Special Supplemental Nutrition Assistance Program for Women, Infants, and Children.

^c^ Some participant households (4/22) had 2 children aged between 2 and 4 years.

^d^Supplemental Nutrition Assistance Program.

### Card Sorting Activity

**Figure 2:**
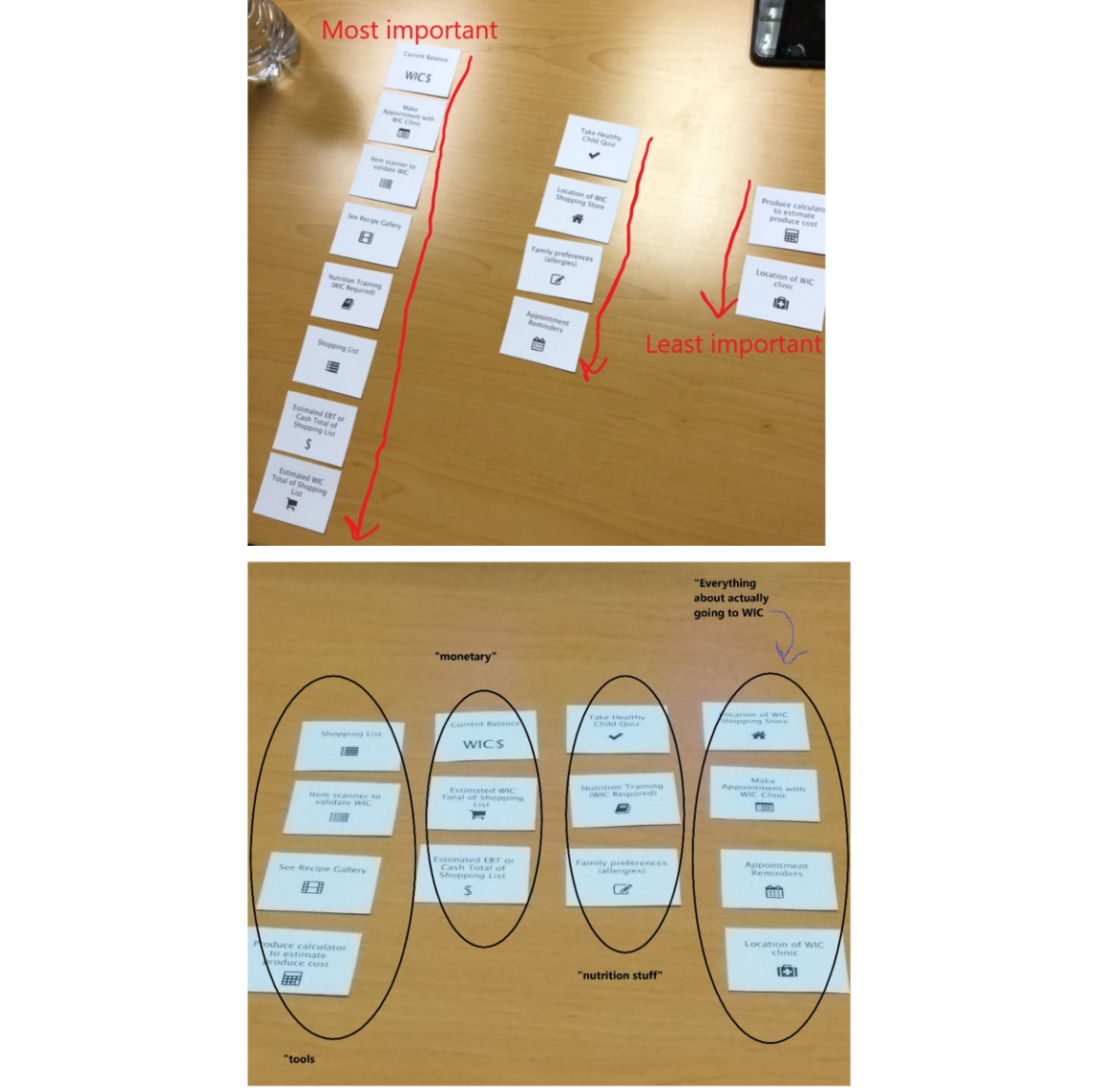
Card sorting activity in progress: sample prioritization (top) and natural groupings (bottom) of potential features in a smartphone app for WIC families by WIC caregivers. WIC: Special Supplemental Nutrition Assistance Program for Women, Infants, and Children.

Among the 22 participants, 4 did not complete the card sorting activity, 1 could not participate owing to literacy issues, and the last 3 participants reviewed these features in an app prototype with these features embedded to review instead of the card sorting activity. Figure 2 depicts the card sorting activity performed by 1 participant. Part way through the study (after 7 participant interviews), 1 app feature card “Estimated EBT or cash total of shopping list” was removed from the card-sorting activity owing to participant confusion.

Most participants (9/22) placed the app features into 3 or 4 natural groupings focused on “clinic/ appointments” (15/22), “shopping/store” (15/22), followed by “education/assessments” (6/22), “location” (4/22), and “recipes/food” (3/22). Some participants (4/22) did not label some of their groupings, whereas others (3/22) did not place the features into any natural groupings.

Prioritization of the app features resulting from the card sorting activity is summarized in Table 2. Card rankings were scored from 1 (highest priority) to 13 (lowest priority), then summarized as sums and means. For the sum and average scores, lower scores represent higher priority, and higher scores represent lower priority. The top 5 features ranked as the highest priority ones were closely tied to their WIC benefits, namely current balance (mean 2.9), item scanner to validate WIC (mean 4.1), make appointment with WIC clinic (mean 4.8), appointment reminders (mean 6.3), and required nutrition training (mean 7.2). The next group of moderately ranked features included shopping list (mean 7.8), location of WIC store (mean 8.0), recipe gallery (mean 8.1), estimated WIC total of shopping list (mean 8.9), and take Healthy Child Quiz (mean 9.1).

**Table 2:** Prioritization of potential features in a smartphone app for Special Supplemental Nutrition Assistance Program for Women, Infants, and Children families by caregivers (N=18).

Rank^a^	App features listed on cards	Sum	Mean
1	Current balance	52	2.9
2	Item scanner to validate WIC^b^	74	4.1
3	Make appointment with WIC Clinic	86	4.8
4	Appointment Reminders	113	6.3
5	Nutrition training (WIC Required)	130	7.2
6	Shopping list	141	7.8
7	Location of WIC shopping store	144	8.0
8	See recipe gallery	146	8.1
9	Estimated WIC total of shopping list	161	8.9
10	Take healthy child quiz	163	9.1

^a^ Card rankings were scored from 1 (highest priority) to 13 (lowest priority). For the sum and mean scores, lower scores represent higher priority, and higher scores represent lower priority.

^b^WIC: Special Supplemental Nutrition Assistance Program for Women, Infants, and Children.

### In-Depth Interview Results

Themes from the qualitative interview portion of the study mainly aligned with and confirmed the results from the card sorting activity. These themes provided deeper insights into the participants’ reasoning, daily activities, and experiences with respect to the WIC program, which guided their opinions about the need for an app and app features designed for their use.

#### Balance Checker

Of the participants who expounded about this feature in the interviews to check their WIC balance (18/22), all described the feature positively saying that it was important, useful, and necessary for keeping track of their benefits.

My current WIC balance would be very important and something I would use all the time.

Breastfeeding WIC Participant, mother of 3: 1 child and 1 infant enrolled in WIC

Most participants were puzzled about how they would keep track of their benefits without an app. However, some (6/22) anticipated how they might try to keep track, citing that they would be able to “call a number” or view it on their receipts or “on a website.” All these participants agreed that these alternatives would be less preferable to a detailed app feature displaying their balance with pictures. Many participants (7/22) described their preferred on-screen appearance of the balance feature, what details should be displayed about the foods, and which items they would want to see at the top of their balance.

Fewer participants expressed as much interest in a subfeature within the balance tracker that would help them calculate their fruit and vegetable balance. Although some described such a feature as potentially helpful (9/22), others (4/22) said the feature might be a “waste of time” or “not very useful” because they either calculated their fruit and vegetable costs themselves, or because it did not really seem important to keep close tabs on the small amount they were allowed ($8-$12 per month) by WIC. Participants (2/10) did describe the difficulty or hassle of trying to tabulate the correct amounts of certain WIC items other than fruits and vegetables (eg, cereal) for going over (or in some cases under) the amount allowed by WIC. Sometimes, participants described choosing cereal that comes in the largest boxes to avoid having to calculate ounces or lose the maximum benefit.

It’s literally like playing, playing cards in the aisle with the kids and other people that are trying to get their cereal too. That's how you end up in the routine of getting the same over and over again. Because you're like ok, I know I can get the big box of (Brand Name A) and the smaller box of (Brand Name B) and just… Let’s just go. So, they don’t get to try the other things. I've literally always wanted to try (Brand Name C) and it's covered (by WIC) and I never incorporate it because the math will be off. I've heard it's like a really good cereal and really good for fiber and things of that nature.

Mother of 2: 1 child enrolled in WIC

Two participants mentioned the desirability of a feature, perhaps within the balance tracker that could show them which cereals and cereal combinations they could choose based on the remaining ounces of cereal in their WIC balance.

#### Scanner to Verify WIC Items

Most participants (17/22) felt a barcode scanner would be useful to verify whether an item is WIC-eligible and available on their WIC balance, especially “in the beginning” of their WIC enrollment. Some (4/22) were already familiar with using barcode scanners in shopping-related apps. Shopping for WIC was described as generally confusing, frustrating, and difficult by most participants (21/22) because WIC items must meet specific size, brand, and nutritional criteria.

Stores lacking labeling or clear labeling of WIC products posed a barrier even for experienced participants (8/22), who stated that stores would change their inventories and WIC-eligible brands. Participants (18/22) described problems at store checkouts including bringing up the wrong item, items not present in the stores' systems, contentious cashiers, length of checkout time, and stigma from other customers. Conflicting information regarding the eligibility of WIC items was a common theme, where participants (10/22) mentioned that their WIC food brochure did not reflect what the store deems eligible or other instances where one store allows an item, whereas another does not.

The study participants described various strategies to make WIC shopping easier, including going to the store at specific non-busy times (2/22), shopping without children (2/22), separating WIC items from the rest of the groceries for a faster check-out (6/22), shopping with someone who has experience with WIC (2/22), asking store employees for help (3/22), and taking photographs of WIC-eligible items at the store to facilitate the next shopping trip (1/22).

The item scanner to validate WIC, that's pretty important because stores are always introducing new things and like I said, even when you have the ounces or you think you know what you're supposed to get on your voucher, it would always be good just to scan it, double-check it before you get up to the counter and then they tell you no, that's not WIC eligible.

Breastfeeding WIC Participant, mother of 3: 1 child and 1 infant enrolled in WIC

Oh, I like that right there- the item scanner to validate WIC- to make sure it is a WIC item. So, like if somebody said no this is not (eligible) then I'm like, “uhuh, watch this then. I'm gonna scan it.

Mother of 2: 2 children enrolled in WIC

#### Clinic-Related Features

Participants (11/22) were enthusiastic about a feature that would allow them to view, make, or change a WIC appointment online via an app. Although participants clearly valued the health benefits and information their families receive through WIC, most (11/22) considered the WIC clinic experience as an inefficient but a necessary burden. Long wait times and difficulties in contacting the WIC clinic (5/22) to enroll and make or reschedule an appointment were recurring themes among the interviewees.

Despite these clinic barriers, one area in which WIC has improved its efficiency over the years is the option for patients to complete the required nutrition education classes online. Most participants said they preferred the online option out of convenience (10/22), although some (2/22) did prefer in-person classes. Participants said they would use an in-app link to meet this requirement, although this feature was not rated high priority unless it allowed them to skip the sign-in process.

#### Shopping List Feature

Of those participants who expounded beyond the card sorting activity (12/22), most (7/12) commented positively about a shopping list feature that would allow them to add WIC and non-WIC items to a shopping list that was linked to their WIC benefits via the app , though some (5/12) remained indifferent to this feature . Most participants (12/22) mentioned that they already use apps or similar features in their smartphones to make shopping lists, whereas others said they prefer to use a pen and paper only for making lists (8/22).

#### Location Features

Participants commented positively (10/22) about a feature that would allow them to find a WIC store, whereas others said the feature was not important, citing that they “already know” where the WIC stores are (one participant even expressed concerns about allowing an app to access her location to enable these features). Participants (7/22) mentioned visiting the same store or stores often. Although proximity was deemed important (8/22), factors other than location were often considered in choosing a WIC store.

Many participants (11/22) said the store environment and customer service were more important than the location because certain stores are often crowded, making it hectic and frustrating to redeem WIC benefits. Participants talked about preferring to go “out of the way” to do their WIC shopping in a less stressful environment (9/22), where they can find better deals (6/22), and where they know that the items will be in stock (1/22).

I think this (WIC store locator) will help because if someone first time will use it he don't know where he will go.

Mother of 5: 1 child and 2 infants (twins) enrolled in WIC

Participants were also asked to provide their opinions about the inclusion of a WIC clinic locator. Although participants mentioned hypothetical situations in which a WIC clinic locator could be useful (eg, first-time patients, when someone moves), no participant regarded a clinic locator as an important feature. Participants said they “know where their WIC clinic is and where they are going.”

However, WIC clinic proximity was regarded as a facilitator to participants' enrollment and ability to attend appointments. Participants (5/22) described the convenience of attending WIC appointments close to their home or at a location that temporarily—but regularly—serves as a “Mobile WIC Clinic” (eg, community center, library). Interviews revealed that in-app notifications about these types of services could serve participants better than a general clinic locator feature.

#### Recipes, Meal Planning, and Dietary Preferences

To gauge interest in a recipe feature within the app, participants were asked to describe their cooking routines. Most participants said they do use recipes (15/22) primarily sourced from the Internet (13/22). However, some participants (inside and outside of the recipe-using group) claimed they were mostly autonomous cookers (8/22) that already “knew how to cook” without relying on recipes for meal ideas.

Many participants described food traditions (10/22), such as learning to cook from a family member or preparing culture-specific foods. However, the most frequently mentioned influence on daily meals was what children wanted to eat (9/22). Health (3/22) and simplicity (3/22) were also factors that participants said influenced their cooking.

Most participants (15/22) said they were interested, would use, or would like to try recipes featured within an app. Some (2/22) mentioned they would not use such a feature, citing that they already knew how to cook what their children liked to eat. Participants who liked the idea of a recipe feature said they would be interested in filtering or pushing recipes based on preparation time (9/22), WIC foods (6/22), keywords (eg, ingredient or recipe name) (5/22), dietary preferences (eg, vegetarian, food allergens) (5/22), kid-friendly recipes (1/22), and meal type (1/22).

Participants (3/22) also mentioned the importance of an attractive layout and photographs within a recipe feature. Although a feature to set dietary preferences and allergies in their family profile was not ranked as high priority, many participants acknowledged that being able to do so would be a “good thing” (10/22).

I would want the recipe gallery because while I’m shopping, if I am trying to be creative and think, “well what else can I get to put with this to actually make a meal?”- go to the quick gallery and you know, see what I already have and you know, see what else I could pick up to make a different meal.

Mother of 2: 1 child enrolled in WIC

I'm visual. If I have a list of food, what to do, yeah- I love to look, and the steps they do. It helps me. I'm like okay. I go back, oh, well I need to put this in. You know? I'm visual. I don't know. Yeah, I like to look, to get pictures, or the video of it.

Mother of 1: 1 child enrolled in WIC

#### Health Assessments and Goal Tracking

All participants expressed that they valued health (either their own, their child's, or their family's), and many participants (7/22) described WIC as a program that promotes health and nutrition. Many of these participants (6/22) expressed interest in receiving health information, tips, and child-feeding advice through the CHEW app. Picky eating was a theme among many participants (11/22) and some expressed a desire to address this issue in their children (6/22) either through the app or otherwise.

Participants were divided on whether they deemed a “child health quiz” as an important feature in the app. Those who said they would use the feature (5/22) described themselves as curious and interested in finding out new information, whereas others (5/22) said the feature was good but not a priority. Others said such a feature would not be something they would use, citing their busy schedules with children and that it was not necessary for redeeming WIC benefits. Certain participants (3/22) said they would consider a quiz feature important if its use counted toward their required WIC nutrition education.

To take healthy child quiz, if they want it to be a priority, they should do it like nutrition training- like required.

Mother of 2: 2 children enrolled in WIC

#### Ancillary Themes Related to App Usefulness

It was apparent from the participant interviews that an app designed to help WIC participants shop should be easier and clearer, as well as contain better information, than existing paper food brochures given at the clinic. Participants (5/22) emphasized that such an app should be straightforward and easy to use.

In general, I just kind of do pretty simple things. Yeah, and I think the pictures is a good way, too (…) make sure the pictures are there. (…) It would make you feel like you're doing something elaborate, but it would be simple and quick, so that's good.

Mother of 1: 1 child enrolled in WIC

Others (6/22) described the existence of language barriers, native languages other than English, and the importance of supporting several languages in the app. A desire to make the app easy to use for those with limited or low literacy was also mentioned (6/22).

Regarding participants’ smartphones and data issues, some (8/22) described barriers to usage, such as having limited data or experiencing slow data speeds. Connecting to available Wi-Fi networks to limit data usage costs was a common strategy for addressing these data issues. Participants mentioned several reasons for deleting an app from their smartphone, the most frequent reasons being infrequent usage (10/22) and limited space/memory (6/22) on their phones. Additional reasons that the participants cited for deleting an app are presented in Table 3.

**Table 3:** Reasons for deleting an app from a smartphone mentioned by Special Supplemental Nutrition Assistance Program for Women, Infants, and Children (WIC) caregivers (N=22) during qualitative interviews about an app for such families.

Reason for deleting an app	Frequency of participant mentions (n)
Do not use it often enough	10
App takes up too much space on phone	6
Children misuse the app	3
App drains battery life of the phone	2
App crashes	2
App is slow to load	1
App is not user friendly/ presents hassles during usage	1

## Discussion

### Principal Results and Comparison With Prior Work

The participants in this study acknowledged the need for a smartphone app with features that would help WIC families keep track of their food benefits and make shopping for WIC easier, especially following the program transition from paper-based food vouchers to EBT cards for redemption of benefits. For the participants in this study, the balance checker and barcode scanner were the most important app features to include in an app for WIC families. This finding confirmed our observations in a previous review [27] of apps and app features for participating WIC families, where these shopping management features were the most commonly present features, and the importance of these features was demonstrated in positive user reviews.

In a previous study that surveyed WIC participants’ current technology usage and preferred methods for interacting with the program, access to the EBT balance (6678/8144, 82%) was reported as highly useful [20]. Nationwide support of EBT for WIC was mandated by 2020 and this technology is not new for some states, although the rollout in most states began within the last 5 years. Wyoming was the earliest adopter of offline EBT for WIC almost 2 decades ago in 2002, whereas New Mexico adopted it in 2007 [23].

A study conducted by the Altarum Institute on behalf of the United States Department of Agriculture (USDA) Economic Research Service that examined the impact of EBT in Kentucky, Michigan, and Nevada (three early adopter states) found positive effects for vendors (less responsibility in policing WIC items for eligibility and quicker receipt of payment) and participants (faster and more discreet checkouts) [25,37]. Focus group participants in the same study expressed that they would like to receive alerts when their benefits are about to expire and would like to access their WIC benefit balance on their smartphones [37]. Similar to the desire for app simplicity and availability in languages other than English among the participants in the current study, the ability to check the WIC balance was notably important for the Spanish-speaking participants in the Altarum study owing to communication barriers with the cashiers when there was an error [37].

The barcode scanning feature was considered important to the participants in this study mainly owing to their negative experiences with WIC item eligibility. Bensley et al also demonstrated that WIC participants would find universal product code (UPC) scanning and verification of WIC items useful (5782/8144, 71%) [20]. Issues at checkout and conflicting information regarding WIC item eligibility remains a barrier to using WIC [13] and is one of the key factors that decrease participants’ perceived value of the program [14]. A barcode scanning feature to verify item eligibility and confirm whether or an item remains on the WIC balance could provide a solution to this common barrier to redeem WIC benefits.

Clinic management features, such as receiving appointment reminders, and viewing, requesting, or making a WIC appointment, were popular options among the participants of his study. In a review of WIC apps, the ability to view an upcoming appointment was an available feature in 8 apps and 3 apps could provide appointment reminders. However, only 1 app at the time of the review provided a feature where participants could request an appointment change through the app [27].

Clinic management features like those outlined above require synchronization with participant information within the clinic’s management information system (MIS) and efforts from the scheduling staff in the clinics. Access to these systems can pose hurdles for third-party providers of digital tools (such as mobile apps) owing to interface design issues, the inability of an older MIS to support secure communication, or WIC agency data security standards [38]. Guidelines from the NWA put forth for WIC agencies wishing to adopt new technologies urges consideration for interoperability between systems or software before adopting or procuring digital tools and mobile apps [38].

In a review of WIC apps available in other states, vendor location or “Find a WIC Store” features were common in 7 WIC apps, and clinic locators were available in 5 apps [27]. Participants in the current study were more enthusiastic about vendor location features than clinic location features, and those that did respond favorably to having a vendor locator did not consider rate it as high priority in the card sorting activity. This result was perhaps due to the participants’ experience with WIC in that none of the participants were new to the program.

Mediocre prioritization of the vendor locator feature may have also been due to the way participants in this study and others did their shopping. Most participants in this study kept their WIC shopping to one or two stores, especially if they received good customer service at a particular store. This finding aligns with Altarum’s EBT transition study [37] that reported participants generally shopped at between one and three major stores.

The clinic locator feature was among the lowest prioritized feature by the participants in this study, which was likely because the participants do not change their home WIC clinic unless they move or choose to recertify elsewhere. Recertification is a lengthy process compared to a typical WIC visit, which has been viewed as a burden by some participants [13]. Participants must recertify their eligibility for WIC every 6-12 months depending on the participant category (eg, pregnancy vs. infant vs. child). If participants’ household incomes fluctuate, placing them in and out of WIC eligibility, they must recertify each time they become eligible for WIC if they desire to remain in the program.

The recipe gallery, meal planning, and dietary preference setting features in a WIC app were received positively in the qualitative interviews but were ranked with medium priority in the card sorting activity among participants in the current study. Searching for and using online recipes were not novel concepts to the participants, especially compared to the shopping management features for WIC. Similar results were found in the study by Bensley et al, where more participants rated the possibility of EBT balance checking and UPC scanning as useful compared to web-based recipes and cooking demos [20].

In a 2018 review of WIC apps, only 2 other apps (WICShopper and Alabama WIC) included a recipe feature. However, user reviews of other apps in the review did voice a desire for a “healthy eating” section [27]. Medium prioritization of this recipe feature in the current study may have also stemmed from the fact that participants were not yet able to see an actual gallery of recipe photographs during the interviews. Healthy recipe ideas and recipes using WIC foods are not new to the WIC program and are often utilized by WIC nutritionists to share with their clientele or posted on local agency websites [39].

NWA newsletters and reports frequently feature testimonials from WIC participants, exclaiming the positive impacts of the WIC program, staff, and services, including healthy recipe ideas [40]. Cooking Matters [41] and EatFresh [42] are resources that gather cooking ideas, recipes, food budgeting, meal planning, and healthy eating information for low-income families online. Incorporating recipes from resources such as these—along with WIC staff knowledge—into a shared space that WIC participants can access through their smartphones could significantly benefit low-income families.

As with the recipes, health assessments and goal tracking features received lower prioritization and enthusiasm compared to shopping management features from our participants. Engagement with the target audience remains a challenge with respect to nutrition education [43]. Introducing an intervention or health tool through a reputable program such as WIC is thus desirable not only for the ability to reach its eligible audiences, but also for the potential impact it can provide to public health [44].

A 2010 workshop summary entitled Planning a WIC Research Agenda cites that most WIC nutrition education research targets the needs of pregnant or postpartum women, and few studies target the needs of preschool children [44]. Other digital tools and apps focusing solely on WIC nutrition education exist [22,30], as do validated web and paper tools targeting preschool children [45,46]. However, delivering nutrition education–based features paired with an app that participants primarily download to help them with their WIC shopping has the potential to impact more participants than a stand-alone tool.

The participants in this study emphasized the importance of delivering a WIC app that is easy to use, hassle free, and available in many spoken languages. Publicly available user reviews of current WIC apps also demonstrate the desire for simplicity, seamlessness, and ease [27]. Although WIC is a federal program, it is administered at the state level, as are the apps available to participants in each state [27]. WIC is multifaceted at the administrative, vendor, and clinic levels, so an app to help participants is only useful if these systems are well integrated, as noted in a recent WIC app study by Zhang et al [29].

In the current study, the participants mentioned the need to delete apps that required too much storage space on their phones or apps that were “slow to load.” Although several low-income households have access to smartphone technology [47], many remain underconnected in that they are only able to access the Internet via their mobile devices [48]. This study highlights many barriers to the successful usage of WIC in its current form and the opportunity to overcome many of these with the transition to an efficient and a useful digital program. However, as revealed by the participant who could not complete the task owing to literacy issues, there might be a risk that some program participants could be left behind owing to low information technology (IT) literacy or limited access to appropriate or suitable devices and the Internet. App developers must be mindful of these issues when designing tools meant to help low-income families.

As our current and ever-changing health care landscape becomes increasingly dependent on technology, the five A’s of access (affordability, availability, accessibility, accommodation, and acceptability) must be considered when implementing these systems to ensure equitable care for those who need it the most [49].

### Limitations

The current study is not without limitations. Owing to the lack of research on apps that reduce the risk factors of childhood obesity among preschool children, only caregivers of WIC children aged 2 to 4 were recruited, which restricted the enrollment of those newer to the program (ie, pregnant women enrolling for the first time or those enrolling with their first infant). This limitation could have altered the prioritization and desire for certain shopping features (eg, scanner or store locator), though this effect was partially remedied by participants’ reflections on the difficulties of being new to the program, compared to navigating the program with experience.

Although all the interview participants spoke English, this was the secondary language for some (6/22) participants in the study. Language barriers that may result from conversing in participants’ secondary language can limit the understanding, intention, and interaction between the interviewer and interviewee. As this study was conducted with WIC families in Tennessee, participant responses may not be empirically generalizable for the WIC population in other states or at the national level.

### Conclusions

By combining in-depth interviewing with a card sorting activity to prioritize features, this study demonstrates how user-centered design can aid the development of an app for low-income families participating in WIC. As WIC continues to modernize, digital tools (such as mobile apps) are becoming increasingly available to streamline WIC services and improve participant experience [38].

The participants in this study were interested in (1) health and nutrition information (they want to eat nutritious food), (2) a modern and graphic-heavy app (eg, something that feels familiar), and (3) app features that increase the accessibility of the program. WIC agencies in states are tasked with procuring and providing digital tools that are user-friendly for participants, supported by staff, affordable, and cost-effective [38].

The following are a summary of recommendations we identified as a result of conducting this study:

- To increase WIC program participation, states should endeavor to work with vendors that provide user-friendly software applications with user-focused features.
- States should attempt to meet users where they are [50] by considering what will drive value for their users, rather than just meeting the requirements of the program.
- Agencies must consider what long-term maintenance and upgrades their digital tools require, as well as their interoperability between systems [38] (eg, EBT vendor or client information systems).
- As WIC is an evidence-based federal nutrition program, WIC agencies should strive to partner with researchers, developers, and vendors that support apps and other digital tools providing avenues to evaluate their potential impact on public health.

## Abbreviations

CHEW: Children Eating Well
EBT: electronic benefits transfer
eWIC: WIC electronic benefits transfer
IT: information technology
MIS: management information system
NWA: National WIC Association
SNAP: Supplemental Nutrition Assistance Program
UPC: universal product code
USDA: United States Department of Agriculture
WIC: Special Supplemental Nutrition Program for Women, Infants, and Children
